# Macronutrient intake is associated with intelligence and neural development in adolescents

**DOI:** 10.3389/fnut.2024.1349738

**Published:** 2024-04-18

**Authors:** Yuko Nakamura, Syudo Yamasaki, Naohiro Okada, Shuntaro Ando, Atsushi Nishida, Kiyoto Kasai, Shinsuke Koike

**Affiliations:** ^1^Center for Evolutionary Cognitive Sciences, Graduate School of Art and Sciences, The University of Tokyo, Tokyo, Japan; ^2^University of Tokyo Institute for Diversity & Adaptation of Human Mind (UTIDAHM), Tokyo, Japan; ^3^Department of Psychiatry and Behavioral Science, Tokyo Metropolitan Institute of Medical Science, Tokyo, Japan; ^4^Department of Neuropsychiatry, Graduate School of Medicine, University of Tokyo, Tokyo, Japan; ^5^International Research Center for Neurointelligence (IRCN), Tokyo, Japan

**Keywords:** macronutrient intake, adolescents, neural development, intelligence, resting-state functional magnetic resonance imaging

## Abstract

**Introduction:**

Macronutrient intake can be one of the most influential factors in cognitive and neural development in adolescents. Adolescence is a specific period of cognitive and neural development, and nutritional effects during this period could be life-long. Therefore, understanding the effects of macronutrient intake on cognitive and neural development in adolescents is crucially important. We thus examined the association across macronutrient intake, intelligence, and neural development using population-based cohort data.

**Methods:**

We conducted two studies. In study 1, we included a total of 1,734 participants (boys, 907, age [mean ± standard deviation] 171.9 ± 3.44 months; range 163.0–186.0 months) from the Tokyo TEEN Cohort (TTC) to examine the association between macronutrient intake and intelligence quotient (IQ). In study 2, we included a total of 63 participants (boys, 38, age 174.4 ± 7.7 months; range 160.7–191.6 months) to investigate the effect of nutrition intake on neural development using graph theory analysis for resting-state functional magnetic resonance imaging (rs-fMRI) derived from a subset of the TTC.

**Results:**

TTC data revealed that a higher IQ was associated in boys with increased protein intake (*β* = 0.068, *p* = 0.031), and in girls, with reduced carbohydrate intake (*β* = −0.076, *p* = 0.024). Graph theory analysis for rs-fMRI at approximately age 12 has shown that impaired local efficiency in the left inferior frontal gyrus was associated with higher carbohydrate and fat intake ([x, y, z] = [−51, 23, 8], *p_FDR-corrected_* = 0.00018 and 0.02290, respectively), whereas increased betweenness centrality in the left middle temporal gyrus was associated with higher carbohydrate, fat, and protein intake ([x, y, z] = [−61, −43, −13], *p_FDR-corrected_* = 0.0027, 0.0029, and 0.00075, respectively). Moreover, we identified a significant moderating effect of fat and protein intake on the relationship between change in *betweenness centrality* over a 2-year measurement gap in the left middle temporal gyrus and intelligence (*β* = 12.41, *p* = 0.0457; *β* = 12.12, *p* = 0.0401, respectively).

**Conclusion:**

Our study showed the association between macronutrient intake and neural development related to intelligence in early adolescents. Appropriate nutritional intake would be a key factor for healthy cognitive and neural development.

## 1 Introduction

During adolescence, the neural network undergoes a fundamental reorganization (1) related to development and maturation of cognitive functions (1, 2). Recent neuroimaging studies have illuminated structural and functional connectivity changes occurring during the transition from childhood to young adulthood, beginning in the primary sensorimotor areas and moving to the higher-order association areas, such as the prefrontal cortex and lateral temporal cortex, that mirror improvement in cognitive abilities (3). In addition, the manifestation of age-related neural reorganization could differ between boys and girls (4), leading to sexual differences in neural and cognitive development (5–7).

Nutrition intake and diet may be one of the most influential factors in neural development (8), cognitive functions, and academic achievement in children (8, 9), among various physical and social environmental factors, such as urbanicity, socio-economic characteristics, and the experience of racism (10). For instance, the effects of several specific nutrients, including omega-3 polyunsaturated fatty acids, iron, and vitamin B12 on brain development and cognitive functions have been shown (11). Research clarifying the effects of macronutrients (such as carbohydrates, fat, and protein) and dietary patterns on development of neural and cognitive functions in children and adolescents has shown that increased fat consumption was significantly associated with decreased hippocampal volume in healthy children (12); the intake of healthier foods (such as whole grains, fish, fruits and/or vegetables) was positively associated with enhanced executive function, whereas that of less-healthy snack foods, sugar-sweetened beverages, and red/processed meats was negatively associated with executive function among children and adolescents (13). In concert with various environmental factors—such as parental aggressiveness or hyperactivity and low education or income status—high fat/sugar intake may contribute to poor inhibitory control (14). A large population-based prospective cohort study showed that high intake of snack and processed foods at age 1 and age 8 years was negatively correlated with global brain volumes at age 10 years, while global brain volumes mediated the relationships between dietary patterns at ages 1 and 8 years and children’s IQ at age 13 years (15). This study indicated that unhealthy dietary patterns such as the western diet—rich in saturated fats and refined carbohydrates—can impair brain development and cognitive functions in children. In addition, a diet high in protein led to improved cognitive ability of children (16, 17). Further, excess body mass—often caused by overconsumption of foods high in fat and sugar (18)—is associated with poor executive functions in children (19). Therefore, macronutrient intake would mediate brain development and cognitive functions in children.

Because intelligence can be defined as the mental ability to integrate various cognitive functions for reasoning, problem solving, and learning (20), different cognitive functions are associated with intelligence. Given this nature of intelligence, various neural circuits are involved in intelligence (20). For instance, a neuroimaging study in children has shown that higher intelligence quotient (IQ) is associated with greater neural connectivity in the attention system, while lower IQ was related to greater neural connectivity in the default mode, emotion, and language systems, after controlling for the effects of age, sex, and age-by-sex interaction (4). Macronutrient intake may influence the neural development underlying intelligence and, given sex differences in neural and cognitive development (4–7), the impact of nutrient intake on this neural development could differ between boys and girls. The adolescent period is a narrow window of neural and cognitive development, during which nutritional effects on neural and cognitive development could be prolonged or life-long. Therefore, while understanding the influence of macronutrient intake on neural development and intelligence in children is critical, research focusing on the relationship between macronutrient intake and neural development or intelligence in children is scarce. Here, we aimed to examine the associations across macronutrient intake, intelligence, and neural development in children aged 12–15 years using an adolescent cohort and resting-state functional magnetic resonance imaging (rs-fMRI) data from subsamples of the cohort.

We hypothesized that—since increased sugar and fat intake were associated with declined cognitive functions (12–14)—higher carbohydrate and fat intake would negatively impact intelligence or neural development associated with intelligence; conversely, given that protein intake was positively related to intelligence (16, 17), higher protein intake would positively impact intelligence or greater neural development. In addition, such impact of macronutrient intake on intelligence or neural development would differ between boys and girls, given sexually determined differences in neural and cognitive development (4, 5, 7).

Among various adaptations to computational techniques for neuroimaging data to capture brain network development (21), we adapted graph theoretical analysis in this study to examine functional connectivity patterns (22). Graph theoretical analysis has recently played a significant role in understanding complex brain connectivity architecture, and can provide access to topological properties that characterize the local and global architecture of the entire brain network connectivity (23). Graph theoretical analysis is thus favorable to examine associations between macronutrient intake and whole-brain neural networks.

## 2 Materials and methods

### 2.1 Study design

We conducted two studies. In study 1, we examined the association between macronutrient intake and intelligence using data from the Tokyo TEEN Cohort (TTC) study, a current prospective population-based cohort study that aims to investigate the developmental trajectory of adolescents (24). We performed study 2 to investigate the effect of nutrition intake on neural development using rs-fMRI data derived from a subset brain imaging cohort [the population-neuroscience study of the TTC (pn-TTC)] (25).

### 2.2 Study 1: associations between macronutrient intake and intelligence

#### 2.2.1 Participants

**Figure 1:**
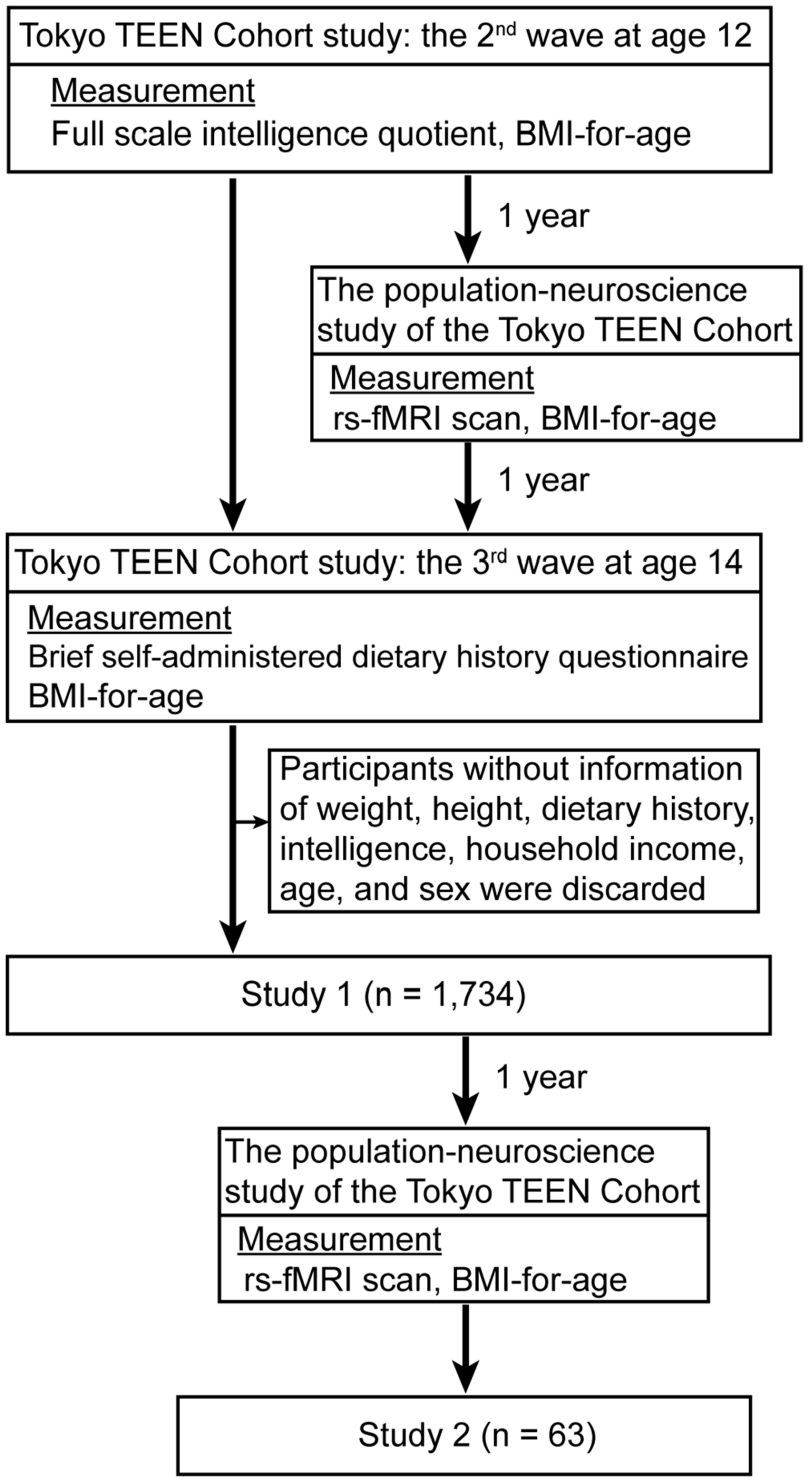
Study protocol of study 1 and 2. For study 1 (*n* = 1,734), we excluded participants for whom data on weight, height, dietary history, intelligence, household income, age, and sex were missing. For study 2 (*n* = 63), we used rs-fMRI data at the 2nd and 3rd waves, excluding participants without data for dietary history, intelligence, household income, age, and sex.

We randomly extracted children born between September 2002 and August 2004 in three local governments in Tokyo (Setagaya, Mitaka, and Chofu) using the Basic Resident Register when the children were 10 years old. Trained interviewers collected data used in this study during home visits when the children were aged 12 (2nd wave) and 14 (3rd wave), achieving a follow-up rate of 94.8% at age 12 (*n* = 3,007) and 84.1% at age 14 (*n* = 2,667). We excluded participants for whom data on weight, height, dietary history, intelligence, household income, age, and sex were missing. Thus, in this study, we included a total of 1,734 participants (boys, 907: girls 827) (Table 1; Figure 1). All procedures were approved by the ethics committees of three research institutes: Tokyo Metropolitan Institute of Medical Science (approval number: 12–35); The University of Tokyo (10057); and SOKENDAI (The Graduate University for Advanced Studies; 2,012,002). We obtained written informed consent from the children’s primary caregivers and informed assent from the children during each wave of the study.

**Table 1:** Demographics of participants in study 1.

	All participants (*n* = 1734) Mean ± S.D. (range)	Boys (*n* = 907) Mean ± S.D. (range)	Girls (*n* = 827) Mean ± S.D. (range)	Sex differences	
				*p*-value	Wilcoxon statistic
Age (months) at 3rd wave	171.89 ± 3.44 (163.00–186.00)	171.87 ± 3.54 (163.00–183.00)	171.91 ± 3.34 (164.00–186.00)	0.47	374,294
BM-for-age (z-value) at 3rd wave	−0.16 ± 0.94 (−3.79–2.90)	−0.21 ± 1.03 (−3.79–2.90)	−0.12 ± 0.82 (−3.16–2.41)	0.009	350,250
Full Scale IQ at 2nd wave	111.26 ± 14.82 (43.79–152.30)	110.31 ± 15.30 (43.79–149.65)	112.30 ± 14.22 (51.82–152.30)	0.003	346,283
Socioeconomic status*	8.12 ± 2.61 (1.00–11.00)	8.04 ± 2.70 (1.00–11.00)	8.21 ± 2.50 (1.00–11.00)	0.41	366,519
Macronutrient intake at 3rd wave					
Carbohydrate (g/day)	350.56 ± 155.95 (66.52–1375.75)	399.49 ± 170.05 (66.52–1375.75)	296.90 ± 117.46 (96.52–1306.08)	1.0	535,141
Fat (g/day)	81.09 ± 30.64 (12.80–278.99)	86.25 ± 32.45 (20.36–278.99)	75.44 ± 27.47 (12.80–237.62)	1.0	454,585
Protein (g/day)	85.48 ± 32.73 (18.94–266.98)	93.23 ± 34.67 (20.31–266.98)	76.98 ± 28.12 (18.94–236.42)	1.0	491,862
Total calorie intake at 3^rd^ wave (kcal/day)	2509.58 ± 950.64 (2333.33–2766.67)	2791.14 ± 1023.12 (569.68–8591.68)	2200.78 ± 751.91 (639.42–7,637)	1.0	520,607

*Socioeconomic status is measured as annual household income divided into 11 categories. 1: 0–990,000 Japanese Yen [JPY]/year, 2: 1,000,000–1,990,000 JPY/year, 3: 2,000,000–2,990,000 JPY/year, 4: 3,000,000–3,990,000 JPY/year, 5: 4,000,000–4,990,000 JPY/year, 6: 5,000,000–5,990,000 JPY/year, 7: 6,000,000–6,990,000 JPY/year, 8: 7,000,000–7,990,000 JPY/year, 9: 8,000,000–8,990,000 JPY/year, 10: 9,000,000–9,990,000 JPY/year, 11: 10,000,000 and more JPY/year. IQ, intelligence quotient; S.D., standard deviation.

#### 2.2.2 Measures

We estimated the children’s general intellectual ability at age 12 by a Full Scale IQ (FIQ) of the Wechsler Intelligence Scale for Children (WISC-III) (26).

We estimated macronutrient and calorie intake at age 14 using a brief self-administered dietary history questionnaire (BDHQ) for Japanese children and adolescents (BDHQ-15y) (27, 28). The BDHQ-15y assesses the quantities of nutrients habitually consumed from foods usually eaten (excluding intake from dietary supplements) as individual units, and is designed to obtain each individual’s nutrient intake, food intake, and information based on a few markers of dietary behaviors. According to a previous longitudinal cohort study, leaving the parental home and completing education were identified as significant factors contributing to changes in diet, including an increase in the consumption of confectionery and sugar-sweetened beverages (29). Thus, it could be assumed that the dietary habits of 12-year-old children (measured for intelligence) and 14-year-old children (measured for dietary habits), who typically live at home and attend compulsory education in Japan, would not differ significantly.

Because socioeconomic status (SES) can influence executive function and brain development in children (30), we assessed annual household income by the self-report questionnaire. Annual household income was divided into 11 categories. 1: 0–990,000 Japanese Yen [JPY]/year, 2: 1,000,000–1,990,000 JPY/year, 3: 2,000,000–2,990,000 JPY/year, 4: 3,000,000 –3,990,000 JPY/year, 5: 4,000,000–4,990,000 JPY/year, 6: 5,000,000–5,990,000 JPY/year, 7: 6,000,000–6,990,000 JPY/year, 8: 7,000,000–7,990,000 JPY/year, 9: 8,000,000–8,990,000 JPY/year, 10: 9,000,000–9,990,000 JPY/year, 11: 10,000,000 and more JPY/year.

At the 2nd and 3rd waves, trained interviewers measured each participant’s height and weight. We then calculated body mass index (BMI) for age (BMI-for-age),—a child’s BMI for their age and sex relative to the reference population, using the WHO criteria (31).

For this study, we did not include parental educational level as a potential confounder because it can be highly correlated with household income, and a previous large cohort study in adolescents (*n* = 3,892 children) has shown that among the socioeconomic factors, including parental education and family income, family income best explains individual differences in cognitive test scores, cortical volume, and thickness (32). In addition, although certain environmental factors such as school type and local environment may influence cognitive development (10), this cohort study randomly selected children from three local governments in Tokyo (Setagaya, Mitaka, and Chofu). Therefore, it is unlikely that there would be significant variation in school type or learning environment within this cohort. As a result, these potential confounding variables were not incorporated into the primary statistical analysis.

#### 2.2.3 Statistics

We performed all statistical analyses using R statistical software (v4.3.0; R Foundation for Statistical Computing, Vienna, Austria). First, we performed Z-score normalization for macronutrient intake, total calorie intake, the measurement gap between FIQ and nutrition intake measures (months), difference between BMI-for-age at age 12 and age 14, SES, and FIQ by subtracting the mean and dividing by the standard deviation. Then, to examine associations between macronutrient intake and intelligence, we performed multiple linear regression analysis using normalized variables. We set the FIQ as a dependent variable, and carbohydrate, fat, and protein intake, as explanatory variables. In addition, we set total calorie intake, the measurement gap between FIQ and nutrition intake measures, difference between BMI-for-age at age 12 and age 14, SES, and sex as potential confounders.

Multiple linear regression analysis assumes that independent variables are not highly correlated with each other, but the variance inflation factor (VIF) for carbohydrate, fat, and protein intake and total calorie intake were 1367.22, 242.36, 69.00, and 3007.77, respectively, showing a high correlation. To address this multicollinearity problem, we created three models—model 1, dependent variable: FIQ, explanatory variable: carbohydrate intake; model 2, dependent variable: FIQ, explanatory variable: fat intake; and model 3, dependent variable: FIQ, explanatory variable: protein intake. All three models also included the measurement gap between FIQ and nutrition intake measures, difference between BMI-for-age at age 12 and at age 14, SES, and sex as potential confounders.

Because sex differences in neural and cognitive development exist during adolescence (4, 5, 7), we performed multiple linear regression analysis using the above three models in boys and girls, separately.

### 2.3 Study 2: associations between macronutrient intake and neural connection

#### 2.3.1 Participants

We subsampled and enrolled some participants from the TTC (25) in study 2. We regarded participants in the TTC who showed interest in the pn-TTC study as candidate participants and enrolled some of them approximately one year later in the pn-TTC study. We used rs-fMRI data at the 2nd and 3rd waves, excluding participants without data for dietary history, intelligence, household income, age, and sex. We included a total of 63 participants (boys, 38: girls 25) in this study (Table 2; Figure 1).

**Table 2:** Demographics of participants in study 2.

	All participants (*n* = 63) Mean ± S.D. (range)	Boys (*n* = 38) Mean ± S.D. (range)	Girls (*n* = 25) Mean ± S.D. (range)	Sex differences	
				*p*-value	Wilcoxon statistic/*t*-value
2nd wave					
Age at rs-fMRI scan (months)	174.41 ± 7.65 (160.68–191.64)	173.63 ± 7.68 (162.36–188.04)	175.60 ± 7.60 (160.68–191.64)	0.32^#^	−1.00^#^
BM-for-age at rs-fMRI scan (z-value)^%^	−0.05 ± 0.87 (−2.12–2.49)	0.09 ± 0.97 (−2.12–2.49)	−0.25 ± 0.68 (−0.98–1.08)	0.95	479
Full Scale IQ	110.84 ± 14.24 (79.52–141.09)	110.68 ± 14.22 (84.77–141.09)	111.07 ± 14.56 (79.52–136.84)	0.92^#^	−0.11^#^
Socioeconomic status*	7.83 ± 2.73 (1.00–11.00)	7.53 ± 2.98 (1.00–11.00)	8.28 ± 2.28 (4.00–11.00)	0.37	412
3rd wave					
Age at rs-fMRI scan (months)	194.25 ± 7.52 (179.40–208.20)	193.81 ± 7.21 (179.88–207.24)	194.91 ± 8.08 (179.40–208.20)	0.58^#^	−0.56^#^
BM-for-age at rs-fMRI scan (z-value)	−0.20 ± 0.96 (−2.83–2.44)	−0.23 ± 1.09 (−2.83–2.44)	−0.14 ± 0.75 (−1.33–1.58)	0.72^#^	−0.35^#^
Macronutrient intake					
Carbohydrate (g/day)	375.22 ± 177.55 (108.79–1302.88)	433.60 ± 197.13 (172.65–1302.88)	286.49 ± 89.08 (108.79–447.99)	1.0	730
Fat (g/day)	87.64 ± 34.00 (30.07–259.89)	95.29 ± 38.49 (51.16–259.89)	76.01 ± 21.65 (30.07–132.73)	1.0	623
Protein (g/day)	92.67 ± 36.01 (34.01–241.60)	102.61 ± 39.13 (55.63–241.60)	77.56 ± 24.39 (34.01–119.46)	1.0	664
Total calorie intake (kcal/day)	2698.34 ± 1104.55 (856.91–8591.68)	3047.65 ± 1225.28 (1430.00–8591.68)	2167.39 ± 591.92 (856.91–3111.21)	1.0	727

*Socioeconomic status is measured as annual household income divided into 11 categories. 1: 0–990,000 Japanese Yen [JPY]/year, 2: 1,000,000–1,990,000 JPY/year, 3: 2,000,000–2,990,000 JPY/year, 4: 3,000,000 –3,990,000 JPY/year, 5: 4,000,000–4,990,000 JPY/year, 6: 5,000,000–5,990,000 JPY/year, 7: 6,000,000–6,990,000 JPY/year, 8: 7,000,000–7,990,000 JPY/year, 9: 8,000,000–8,990,000 JPY/year, 10: 9,000,000–9,990,000 JPY/year, 11: 10,000,000 and more JPY/year. ^#^Statistical results of Student’s *t*-test. ^%^Seven participants do not have BMI-for-age data. IQ, intelligence quotient, S.D., standard deviation.

#### 2.3.2 Magnetic resonance imaging data acquisition

All participants underwent rs-fMRI sessions at the 2nd and the 3rd waves. We instructed them to remain still and gaze at a black cross on a white screen during the scanning, for one run (10 min 10 s) at the 2nd wave, and four runs (5 min 36 s each) at the 3rd wave.

We acquired all MR images using a MAGNETOM Prisma 3.0 Tesla scanner (Siemens Healthineers, Erlangen, Germany). At the 2nd wave, we used a 64-channel head coil and acquired anatomical images using a T1-weighted protocol (repetition time (TR) = 1900 ms, echo time (TE) = 2.53 ms, flip angle (FA) = 9°, field-of-view (FOV) = 256 × 256 mm^2^, and resolution 1.0 × 1.0 × 1.0 mm^3^). For functional images, we acquired T2*-weighted images using gradient-echo echo-planar imaging (EPI) (TR = 2,500 ms, TE = 30 ms, 38 slices, FA = 80°, FOV = 212 × 212 mm^2^, and resolution = 3.3 × 3.3 × 4.0 mm^3^) in ascending order. At the 3rd wave, we used a 32-channel head coil and acquired anatomical images using a T1-weighted protocol (TR = 2,400 ms, TE = 2.22 ms, FA = 8°, FOV = 256 × 256 mm^2^, resolution 0.8 × 0.8 × 0.8 mm^3^). For functional images, we acquired T2*-weighted images using EPI with a multiband acceleration factor 8 and generalized, auto-calibrating (TR = 800 ms, TE = 37 ms, 72 slices, FA = 52°, FOV = 208 × 208 mm^2^, and resolution = 2.0 × 2.0 × 2.0 mm^3^) using an interleaved manner to reduce the cross-talk of the slice selection pulse.

#### 2.3.3 Image preprocessing

We preprocessed all rs-fMRI data using Statistical Parametric Mapping 12 (SPM12) software with the CONN functional connectivity toolbox (CONN, version 22a) (33). For rs-fMRI data at the 2nd and 3rd waves, we performed conventional preprocessing including slice-timing correction, realignment and unwarping, denoising, normalization (onto the standard Montreal Neurological Institute and Hospital space), and smoothing (6-mm full-width at half-maximum Gaussian filter) using the default parameter settings of SPM12. We estimated six motion parameters (three rotation and three translation parameters) during the realignment step. We then used the Artifact Detection Tools toolbox implemented in CONN to detect potential outlier scans identified from the observed global blood-oxygen-level-dependent (BOLD) signal and the amount of participant motion. We flagged acquisitions with framewise displacement greater than 0.9 mm or global BOLD signal changes greater than 5 S.D.s as potential outliers.

#### 2.3.4 Graph theory analyses

We performed graph theory analyses using the default functional connectivity processing pipeline in the CONN toolbox. We first cleaned the preprocessed fMRI data using the CONN functional connectivity toolbox denoising pipeline. This included ART-based motion scrubbing, outlier volume removal, regression of white matter and cerebrospinal fluid signals using the component-based noise correction method (CompCor) strategy (34), band-pass temporal filtering (*f* = 0.007–0.1 Hz), and linear detrending. We derived the regions of interest (ROIs) used here from a freely available ROI atlas (35) which included 200 ROIs generated by a gradient-weighted Markov Random Field model that could parcelize neural regions representing neurobiologically meaningful features of brain organization using rs-fMRI data from 1,489 participants. We treated each ROI as a “node” within a whole-brain network. We extracted the BOLD time course for each ROI for each participant to create a graph adjacency matrix. First, for all pairs of ROIs, we created an ROI-to-ROI Correlation (RRC) matrix ( γ) using an extracted time course. We then formed a graph adjacency matrix by thresholding the RRC matrix and retaining 15% of the strongest positive correlations; thus, in each participant graph, 15% of all possible edges were represented. These graphs were undirected in that the association between regions was bidirectional. From the resulting graphs, we computed a number of measures addressing topological properties of each ROI within the graph. Therefore, at the second level, we adapted the CONN toolbox’s automated graph theory analysis algorithms^1^ to explore associations between graph theoretic metrics and macronutrient intake using rs-fMRI from the 2nd wave, controlling for sex and SES using a false discovery rate (FDR)-corrected *p*-value threshold of *p* < 0.05 (all reported *p*-values are two-tailed). We performed the same analysis in boys and girls separately controlling for SES.

We measured the following graph theoretic metrics: *Betweenness Centrality*—a centrality measure of a ROI that acts as a bridge along the shortest path between two other ROIs; *Local Efficiency*— a measure of local integration or coherence, characterizing the degree of inter-connectedness among all nodes within a node neighboring sub-graph; *Eigenvector Centrality*—the influence of a ROI based on its connectedness with other high-scoring nodes in a network; *Degree and Cost* at each ROI—measures of network centrality, characterizing the degree of local connectedness of each ROI within a graph; *Average Path Distance*—a measure of node centrality within a network, characterizing the degree of global connectedness of each ROI within a graph; *Clustering Coefficient*— a measure of the degree to which nodes in a graph tend to cluster together; *Global Efficiency* at a node—a measure of this node centrality within the network, characterizing the degree of global connectedness of each ROI; *Eccentricity*—the maximum distance between an ROI and all other ROIs. Each of these statistics emphasizes a distinct aspect of the nodes’ varying roles in organizing information processing across the brain [for a review of these graph theory metrics, please see (23)].

#### 2.3.5 Associations across intelligence, macronutrient intake, and neural development

To investigate not only the effect of neural development and macronutrient intake on intelligence but also the potential moderating effect of macronutrient intake on the relationship between neural development and intelligence, we performed moderation analysis using R statistical software (v4.3.0; R Foundation for Statistical Computing, Vienna, Austria). First, regarding the ROIs that showed significant associations with nutrient intake at the 2nd wave, we calculated changes in ROI-specific graph theoretic metrics from the 2nd to the 3rd waves and used them as neural development. We then performed a multiple regression model in which we set FIQ as the dependent variable, and macronutrient intake, neural development, and an interaction term for neural development and macronutrient intake as explanatory variables. We included sex in this model as a potential confounder only when considering all participants, but not when considering boys only or girls only.

## 3 Results

We performed two studies: Study 1 aimed to examine the association between macronutrient intake and intelligence using large adolescent cohort data (TTC), and Study 2 aimed to detect the effect of nutrition intake on neural development using rs-fMRI data derived from a subset TTC.

### 3.1 Study 1: macronutrient intake and intelligence

Multiple linear regression was performed to examine associations between macronutrient intake and intelligence. Multiple linear regression showed a positive association between protein intake and FIQ [beta coefficient (*β*) = 0.55, *p* = 0.004], a negative association between total calorie intake and FIQ (*β* = −2.51, *p* = 0.046), and a positive association between SES and FIQ (*β* = 0.29, *p* < 0.001). Carbohydrate and fat intake were not significantly associated with FIQ (*β* = 1.57, *p* = 0.062; *β* = 0.61, *p* = 0.085). The measurement gap between FIQ and nutrition intake measures, difference in BMI-for-age at age 12 and 14, and sex were not significantly associated with FIQ (*ps* > 0.090).

VIF for carbohydrate, fat, protein, and total calorie intake were 1367.22, 242.36, 69.00, and 3007.77, respectively, indicating high correlation. To address this multicollinearity problem, we tested associations between FIQ and each macronutrient separately. However, we found no significant association between FIQ and carbohydrate, fat, or protein intake (*β* = −0.029, *p* = 0.233; *β* = 0.005, *p* = 0.845; *β* = 0.029, *p* = 0.216, respectively) (Table 3).

**Table 3:** Multiple linear regression models describing the association between Full Scale IQ scores at the 2nd wave and macronutrient intakes at the 3rd wave.

Dependent variable	Explanatory variables [beta coefficient (*β*), *p*-value]				
All participants					
Model 1					
FIQ	Carbohydrate intake	The measurement gap between FIQ and nutrition intake measures	Difference between BMI-for-age at age 12 and age 14	SES	sex
	*β* = −0.029, *p* = 0.233	*β* = −0.007, *p* = 0.766	*β* = 0.003, *p* = 0.915	*β* = 0.292, *p* < 0.001	*β* = 0.095, *p* = 0.057
Model 2					
FIQ	Fat intake	The measurement gap between FIQ and nutrition intake measures	Difference between BMI-for-age at age 12 and age 14	SES	sex
	*β* = 0.005, *p* = 0.845	*β* = −0.006, *p* = 0.784	*β* = 0.001, *p* = 0.972	*β* = 0.292, *p* < 0.001	*β* = 0.116, *p* = 0.015
Model 3					
FIQ	Protein intake	The measurement gap between FIQ and nutrition intake measures	Difference between BMI-for-age at age 12 and age 14	SES	sex
	*β* = 0.029, *p* = 0.216	*β* = −0.005, *p* = 0.819	*β* < 0.001, *p* = 1.00	*β* = 0.291, *p* < 0.001	*β* = 0.130, *p* = 0.008
Boys					
Model 1					
FIQ	Carbohydrate intake	The measurement gap between FIQ and nutrition intake measures	Difference between BMI-for-age at age 12 and age 14	SES	
	*β* < −0.001, *p* = 0.993	*β* = 0.004, *p* = 0.895	*β* = 0.002, *p* = 0.960	*β* = 0.313, *p* < 0.001	
Model 2					
FIQ	Fat intake	The measurement gap between FIQ and nutrition intake measures	Difference between BMI-for-age at age 12 and age 14	SES	
	*β* = 0.036, *p* = 0.251	*β* = 0.005, *p* = 0.878	*β* < 0.001, *p* = 0.994	*β* = 0.312, *p* < 0.001	
Model 3					
FIQ	Protein intake	The measurement gap between FIQ and nutrition intake measures	Difference between BMI-for-age at age 12 and age 14	SES	
	*β* = 0.068, *p* = 0.031	*β* = 0.006, *p* = 0.848	*β* = −0.001, *p* = 0 0.973	*β* = 0.311, *p* < 0.001	
Girls					
Model 1					
FIQ	Carbohydrate intake	The measurement gap between FIQ and nutrition intake measures	Difference between BMI-for-age at age 12 and age 14	SES	
	*β* = −0.076, *p* = 0.024	*β* = −0.023, *p* = 0.503	*β* = 0.005, *p* = 0.888	*β* = 0.266, *p* < 0.001	
Model 2					
FIQ	Fat intake	The measurement gap between FIQ and nutrition intake measures	Difference in BMI-for-age between age at 12 to 14	SES	
	*β* = −0.040, *p* = 0.235	*β* = −0.022, *p* = 0.516	*β* = 0.001, *p* = 0.965	*β* = 0.267, *p* < 0.001	
Model 3					
FIQ	Protein intake	The measurement gap between FIQ and nutrition intake measures	Difference between BMI-for-age at age 12 and age 14	SES	
	*β* = −0.029, *p* = 0.387	*β* = −0.022, *p* = 0.522	*β* = 0.001, *p* = 0.971	*β* = 0.267, *p* < 0.001	

BMI, Body mass index; FIQ, Full scale intelligence quotient; SES, Socioeconomic status.

FIQ in boys was positively associated with protein intake (*β* = 0.068, *p* = 0.031), but not with carbohydrate (*β* < −0.001, *p* = 0.993) or fat intake (*β* = 0.036, *p* = 0.251) (Table 3).

FIQ in girls was negatively associated with carbohydrate intake (*β* = −0.076, *p* = 0.024), but not with protein (*β* = −0.029, *p* = 0.387) or fat intake (*β* = −0.040, *p* = 0.235) (Table 3).

### 3.2 Study 2: macronutrient intake and brain networks

**Figure 2:**
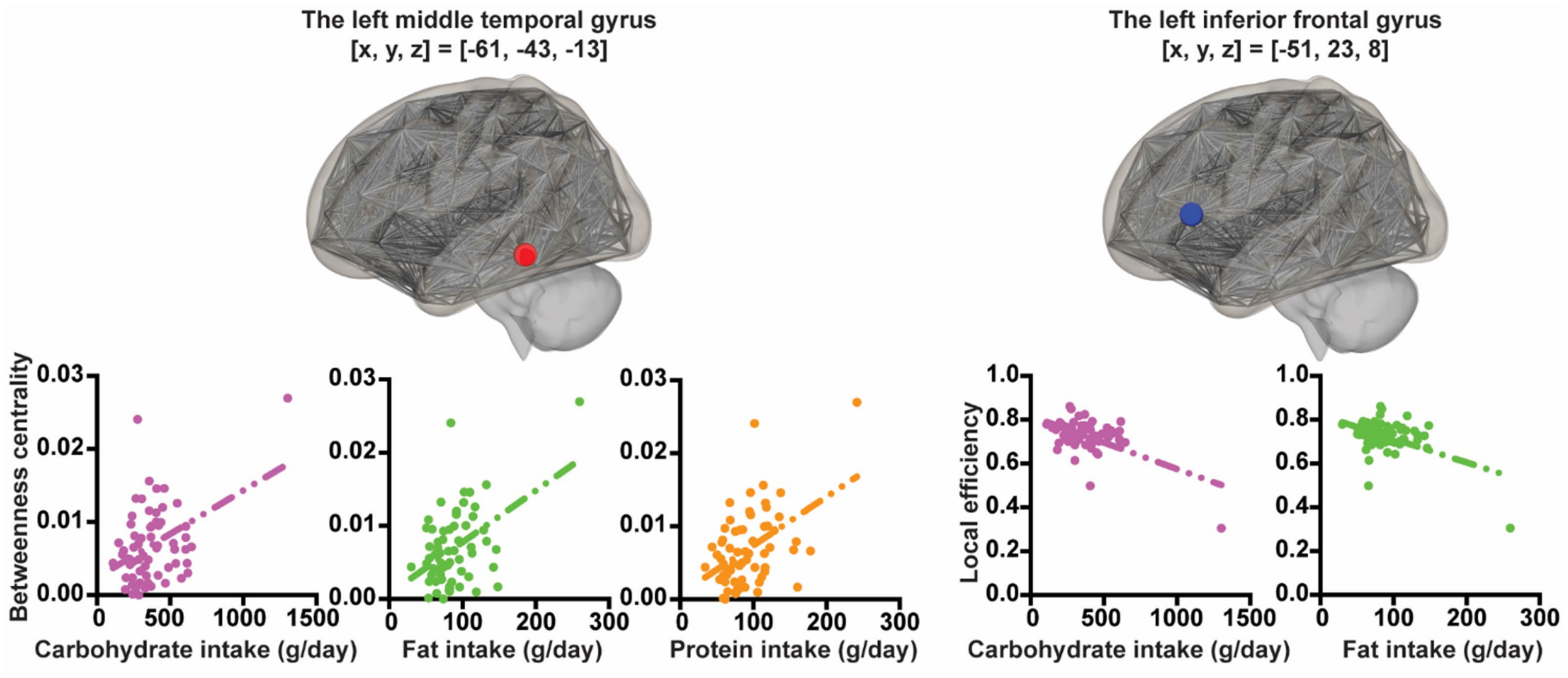
The associations between nutrient intake and graph theoretic metrics. The *X*-axis of the plot represents nutrition intake. The *Y*-axis of the plot represents graph theoretic metrics.

Using rs-fMRI at the 2nd wave, associations between several graph theory metrics for ROIs and each macronutrient intake were explored controlling for sex and SES. In all participants, greater carbohydrate intake was associated with less local efficiency in the left triangular part of the inferior frontal gyrus ([x, y, z] = [−51, 23, 8], *t* = −5.49, *p_FDR-corrected_* = 0.00018) and with greater betweenness centrality in the left middle temporal gyrus ([x, y, z] = [−61, −43, −13], *t* = 4.75, *p_FDR-corrected_* = 0.0027). Greater fat intake was associated with less local efficiency in the left triangular part of the inferior frontal gyrus ([x, y, z] = [−51, 23, 8], *t* = −4.13, *p_FDR-corrected_* = 0.02290) and with greater betweenness centrality in the left middle temporal gyrus ([x, y, z] = [−61, −43, −13], *t* = 4.72, *p_FDR-corrected_* = 0.0029). Greater protein intake was associated with greater betweenness centrality in the left middle temporal gyrus ([x, y, z] = [−61, −43, −13], *t* = 5.10, *p_FDR-corrected_* = 0.00075) (Figure 2).

In boys, after statistically controlling for SES, greater carbohydrate intake was associated with less local efficiency in the left triangular part of the inferior frontal gyrus ([x, y, z] = [−51, 23, 8], *t* = −4.77, *p_FDR-corrected_* = 0.0063) and with greater betweenness centrality in the left middle temporal gyrus ([x, y, z] = [−61, −43, −13], *t* = 4.95, *p_FDR-corrected_* = 0.0037). Greater fat intake was associated with greater betweenness centrality in the left middle temporal gyrus ([x, y, z] = [−61, −43, −13], *t* = 4.15, *p_FDR-corrected_* = 0.0404) and with less eccentricity in the left posterior cingulate gyrus (brodmann area[BA] 23) ([x, y, z] = [−5, −29, 27], *t* = −4.44, *p_FDR-corrected_* = 0.0173) and the right precuneus ([x, y, z] = [11, −74, 25], *t* = −4.05, *p_FDR-corrected_* = 0.0270). Greater protein intake was associated with greater betweenness centrality in the left middle temporal gyrus ([x, y, z] = [−61, −43, −13], *t* = 4.71, *p_FDR-corrected_* = 0.0187).

In girls, after statistically controlling for SES, we found no significant associations between graph theory metrics and each macronutrient intake.

### 3.3 Study 2: associations across macronutrient intake, neural development, and intelligence

**Figure 3:**
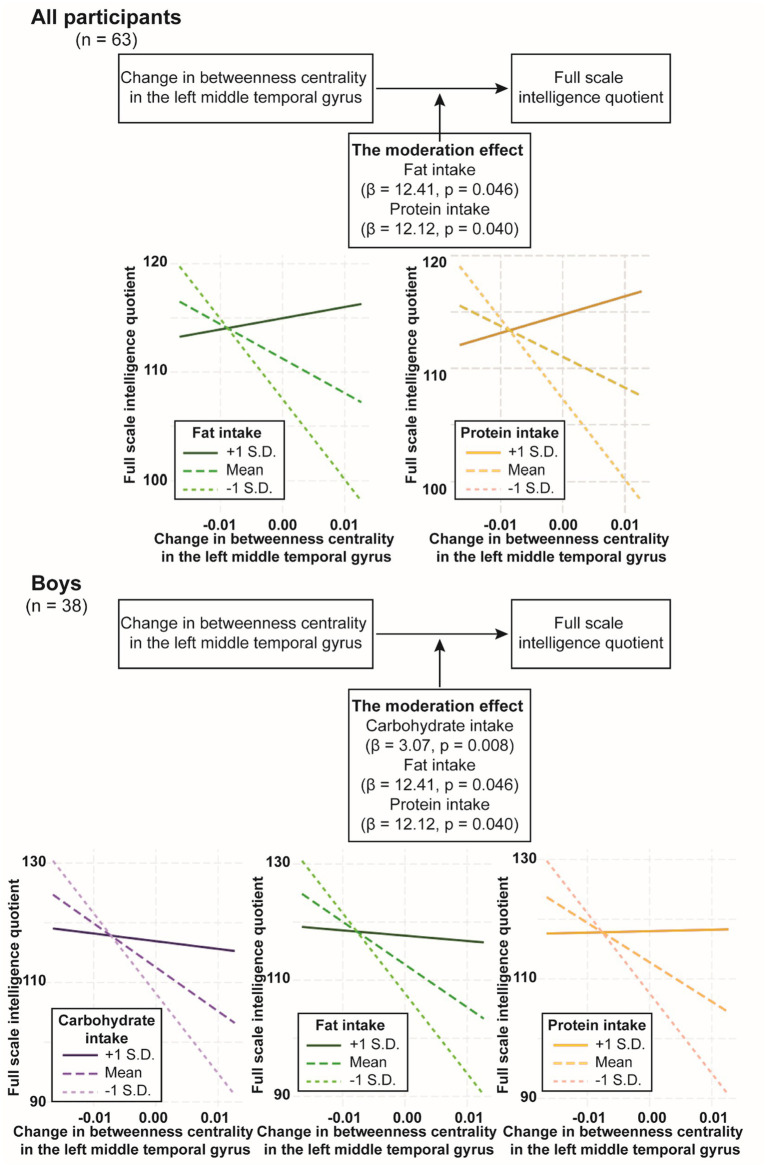
The moderation effect of nutrition intake on the relationship between full scale intelligence quotient change in betweenness centrality in the left middle temporal gyrus. The diagram depicts the moderation effect on the relationship between full scale intelligence quotient and change in betweenness centrality in the left middle temporal gyrus. The *X*-axis of the plot represents the independent variable, which is full scale intelligence quotient. The *Y*-axis of the plot represents the dependent variable, which is change in betweenness centrality in the left middle temporal gyrus. The lines on the plot represent different levels of the moderator variable. Each line’s slope illustrates the relationship between full scale intelligence quotient and change in betweenness centrality in the left middle temporal gyrus at that specific level of the moderator variable. *β*, beta coefficient; S.D., standard deviation.

Moderation analysis was conducted using rs-fMRI at waves 2 and 3 to examine the effects of neural development and macronutrient intake on intelligence and the potential moderating effect of macronutrient intake on the relationship between neural development and intelligence. In all participants, we found no significant moderation effect of carbohydrate intake on the relationship between intelligence and change in local efficiency in the left triangular part of the inferior frontal gyrus or change in betweenness centrality in the left middle temporal gyrus (*p* = 0.294 and *p* = 0.1093, respectively). Likewise, we found no significant moderation effect of fat intake on the relationship between intelligence and change in local efficiency in the left triangular part of the inferior frontal gyrus (*p* = 0.26), but contrastingly, the moderation effect of fat intake on the relationship between intelligence and change in betweenness centrality in the left middle temporal gyrus was significant (*β* = 12.41, *p* = 0.0457) (Figure 3; Table 4). Similarly, the moderation effect of protein intake on the relationship between intelligence and change in betweenness centrality in the left middle temporal gyrus was significant (*β* = 12.12, *p* = 0.0401) (Figure 3; Table 4).

**Table 4:** Associations across macronutrient intake, neural development, and intelligence.

Dependent variable	Explanatory variables [beta coefficient (*β*), *p*-value]			
All participants				
FIQ	The moderation effect of carbohydrate intake	Carbohydrate intake	Change in betweenness centrality in the left middle temporal gyrus	Sex
	*β* = 1.792, *p* = 0.109	*β* = 0.012, *p* = 0.393	*β* = −1,001, *p* = 0.089	*β* = 0.252, *p* = 0.954
FIQ	The moderation effect of fat intake	Fat intake	Change in betweenness centrality in the left middle temporal gyrus	Sex
	*β* = 12.41, *p* = 0.046	*β* = 0.110, *p* = 0.132	*β* = −1,406, *p* = 0.046	*β* = 0.482, *p* = 0.905
FIQ	The moderation effect of protein intake	Protein intake	Change in betweenness centrality in the left middle temporal gyrus	Sex
	*β* = 12.12, *p* = 0.040	*β* = 0.104, *p* = 0.094	*β* = −1,397, *p* = 0.055	*β* = 1.184, *p* = 0.776
FIQ	The moderation effect of carbohydrate intake	Carbohydrate intake	Change in local efficiency in the left inferior frontal gyrus	Sex
	*β* = −0.065, *p* = 0.294	*β* = 0.014, *p* = 0.397	*β* = 38.948, *p* = 0.421	*β* = 1.848, *p* = 0.664
FIQ	The moderation effect of fat intake	Fat intake	Change in local efficiency in the left inferior frontal gyrus	Sex
	*β* = −0.347, *p* = 0.261	*β* = 0.096, *p* = 0.212	*β* = 38.425, *p* = 0.447	*β* = 1.600, *p* = 0.683
Boys				
FIQ	The moderation effect of carbohydrate intake	Carbohydrate intake	Change in betweenness centrality in the left middle temporal gyrus	
	*β* = 3.074, *p* = 0.008	*β* = 0.02217, *p* = 0.118	*β* = −2068, *p* = 0.003	
FIQ	The moderation effect of fat intake	Fat intake	Change in betweenness centrality in the left middle temporal gyrus	
	*β* = 16.72, *p* = 0.008	*β* = 0.129, *p* = 0.085	*β* = −2,328, *p* = 0.003	
FIQ	The moderation effect of protein intake	Protein intake	Change in betweenness centrality in the left middle temporal gyrus	
	*β* = 17.45, *p* = 0.003	*β* = 0.133, *p* = 0.035	*β* = −2,449, *p* = 0.002	
FIQ	The moderation effect of carbohydrate intake	Carbohydrate intake	Change in local efficiency in the left inferior frontal gyrus	
	*β* = −0.110, *p* = 0.109	*β* = 0.030, *p* = 0.101	*β* = 63.176, *p* = 0.261	
FIQ	The moderation effect of fat intake	Fat intake	Change in eccentricity in the left posterior cingulate gyrus	
	*β* = −0.039, *p* = 0.877	*β* = 0.060, *p* = 0.349	*β* = 2.695, *p* = 0.904	
FIQ	The moderation effect of fat intake	Fat intake	Change in eccentricity in the right precuneus	
	*β* = −0.078, *p* = 0.621	*β* = 0.058, *p* = 0.346	*β* = 14.76, *p* = 0.387	

FIQ, Full scale intelligence quotient.

In boys, the moderation effect of carbohydrate intake on the relationship between intelligence and change in local efficiency in the left triangular part of the inferior frontal gyrus was not significant (*p* = 0.109), whereas the moderation effect of carbohydrate intake on the relationship between intelligence and change in betweenness centrality in the left middle temporal gyrus was significant (*β* = 3.074, *p* = 0.008) (Figure 3; Table 4). The moderation effect of fat intake on the relationships between intelligence and change in local efficiency in the left triangular part of the inferior frontal gyrus, or change in eccentricity in the left posterior cingulate gyrus or in the right precuneus were not significant (*ps* > 0.261), whereas the moderation effect of fat intake on the relationship between intelligence and change in betweenness centrality in the left middle temporal gyrus was significant (*β* = 16.72, *p* = 0.008) (Figure 3; Table 4). The moderation effect of protein intake on the relationship between intelligence and change in betweenness centrality in the left middle temporal gyrus was significant (*β* = 17.45, *p* = 0.003) (Figure 3; Table 4).

## 4 Discussion

Here we aimed to examine the impact of macronutrient intake on the association between intelligence and neural development in adolescents. Using a large adolescent cohort, we found that higher general intelligence was associated with increased protein intake in boys, and in girls, with lower carbohydrate intake. In addition, rs-fMRI data showed that lower carbohydrate and fat intake were associated with higher inter-connectedness in the left triangular part of the inferior frontal gyrus, and all macronutrient intake was positively correlated with greater centrality in the left temporal gyrus in all participants. Also, in boys, lower carbohydrate intake was associated with greater inter-connectedness in the left triangular part of the inferior frontal gyrus, whereas all macronutrient intake was positively correlated with greater centrality in the left temporal gyrus. Additionally, in boys, higher fat intake was associated with lower long-range connectivity in the left post cingulate gyrus and right precuneus. Moreover, in boys, but not girls, carbohydrate, fat, and protein intake moderated the relationship between general intelligence and neural development in the left middle temporal gyrus, indicating that the impact of neural development in the middle temporal gyrus on general intelligence differs depending on the level of macronutrient intake. Generally, in accordance with our hypothesis, protein intake was positively associated with higher general intelligence, while carbohydrate and fat intake were negatively associated with general intelligence and neural connectivity. These associations between macronutrient intake and intelligence or neural connectivity varied depending on sex.

In line with the hypothesis, higher protein intake was positively associated with greater intelligence in boys, partially replicating previous studies. Wang and colleagues reported a high protein dietary pattern in children aged 10–15 years was associated with greater mathematical ability after controlling for sex, age, nationality, household registration, school type, parental education level, family learning environment, annual household income, and family size (16). Another study showed a positive association in children aged 6–8 years between protein intake and higher cognitive function related to attention after controlling for age (17). Thus, protein intake during adolescence would facilitate general intelligence in boys.

As we expected, higher carbohydrate intake was negatively associated with general intelligence in girls. A previous study demonstrated that an unhealthy dietary pattern, such as the western diet, was connected with impaired brain development and lower IQ in early adolescents (15). In addition, another study with greater sample size (*n* = 17,348 school children) showed the connection between more frequent high-calorie food intake and children’s weaker academic achievement (9). Therefore, in line with these results, our findings suggest that in girls, higher carbohydrate intake would be connected with lower intelligence.

Brain imaging data showed that, in all participants and boys, higher carbohydrate intake was negatively associated with lower local efficiency in the left triangular part of the inferior frontal gyrus. The triangular part of the inferior frontal gyrus acts as the hub that links the sensory/somatomotor network, the default mode network (DMN), and the dorsal and ventral attention network (36). This region is also involved in verbal and non-verbal context recognition (37), language processing (38), and unconscious information processing (39). Therefore, higher local efficiency in this region would indicate effective information processing—such as passing information through various brain networks, including the sensory/somatomotor network, DMN, and dorsal and ventral attention network—to facilitate an understanding of various contexts, which is a cognitive function implicated in intelligence (20, 40). The current study found a negative association between carbohydrate and fat intake and local efficiency in the triangular part of the inferior frontal gyrus. Additionally, carbohydrate intake was negatively associated with intelligence in girls. These findings suggest that excessive habitual carbohydrate intake may impair local efficiency in the triangular part of the inferior frontal gyrus and have a negative impact on intelligence in children, or at least girls.

We found that higher fat intake was linked to lower long-range connectivity in the left post cingulate gyrus and right precuneus in boys. The post cingulate gyrus (BA 23) is a part of the frontoparietal control network (38) and the DMN (41), constituting a core hub in the human connectome (42), with a relatively large-scale neural network (43), and is involved in various cognitive functions, including memory, spatial navigation, self-reflection and self-imagery, and decision making (44). The precuneus, involved in the visual field (38) and the DMN (45), has a large-scale neural network (46). Serving as a global hub, it is likely to be integrated in early adolescence (4). This region has a role in facilitating successful episodic memory retrieval (47). For instance, a previous fMRI study demonstrated the activation of this region during memory retrieval tasks (48). Further, the precuneus could be involved in executive functions, such as cognitive flexibility (49). Thus, long-range connectivity with the precuneus is related to various cognitive functions. Collectively, the post cingulate gyrus and precuneus have a long-range connectivity and play various roles in intelligence. Long-range functional connectivity—rather than short-range functional connectivity—could contribute to IQ (50). In addition, higher fat and sugar intake could be connected with attenuated IQ (12, 13, 15). As demonstrated by the current results, long-range connectivity in the post cingulate gyrus and precuneus were negatively associated with fat intake in boys; fat intake would negatively impact developing long-range functional connectivity in the post cingulate gyrus and precuneus and be connected with lower intelligence.

Brain imaging data also showed that, at the 2nd wave, greater intake of carbohydrate, fat, and protein was positively associated with greater betweenness centrality in the left middle temporal gyrus in all participants. Moreover, the moderation analysis revealed that the impact of neural development in betweenness centrality in the middle temporal gyrus on intelligence differs depending on the level of the moderation effect of carbohydrate, fat, and protein intake in boys, and the moderation effect of fat and protein intake in all participants. The middle temporal gyrus is a part of the frontoparietal control network (38) and involved in the regulation of perceptual attention (51), and also seems to be a part of the traditional sensory language area (52). Further, the region could be associated with the DMN (53) and creative ability (54). The middle temporal gyrus is thus involved in various cognitive functions related to intelligence. Given that the ROI with higher betweenness centrality more frequently lies on the shortest paths between other ROIs, greater betweenness centrality in the left middle temporal gyrus would suggest greater integrated functional connectivity within this region. The current results, consistent with previous studies (16, 17), suggest that higher protein intake in boys is associated with increased IQ. This supports our hypothesis that protein intake moderates connectivity integration in the middle temporal gyrus during development and promotes general intelligence.

The relationship between macronutrient intake and general intelligence, as well as the moderating effect of macronutrient intake on the relationship between neural development and general intelligence, are influenced by sex, which is consistent with our hypothesis. In a previous large-cohort study, sex differences in multiple cognitive functions appeared during the progression from early teens to late teens (5), and could be connected with brain maturation. Gray matter and white matter volumes in the frontal, temporal, and parietal regions show the effect of interaction between age and sex in adolescents (5). The functional and structural connectivity in the post cingulate gyrus also mediates sex differences in cognitive functions (6). Further, compared to boys, girls show earlier white matter development, and such sex difference influences cognitive performance (7). Thus, earlier brain maturation in girls is associated with their earlier cognitive development. Moreover, significant effects of sex, IQ, and age interactions on microstructures of the white matter were seen in frontoparietal areas (55). In the current study, only boys showed moderation effects of macronutrient intake on the association between IQ and neural development in the middle temporal gyrus, a part of the frontoparietal control network. These results suggest that neural development in boys at mid-teens is susceptible to macronutrient intake because of late maturation in the middle temporal gyrus. Given the link between structural and functional brain development and the surge of gonadal hormones (5), sex differences in neural and cognitive development may be attributed to gonadal hormones, in concert with which other factors—such as social and cultural factors (56)—may also contribute to such sex differences,. Further studies are needed to elucidate the cause of sex differences in neural and cognitive development.

Certain limitations should be considered in interpreting the current findings. First, although we included socioeconomic status, BMI-for-age, and sex as confounders, we did not include other potential confounders that may affect intelligence or neural development, such as environmental factors (10). Future studies should consider including these potential confounders to examine the effect of nutrition intake on intelligence or neural development in adolescents. Second, the number of participants for the imaging study and moderation analysis would not be adequate. Suitable sample sizes for functional neuroimaging studies using graph theory analysis have been debated, and a group size of approximately 20 was suggested as sufficient (57). Thus, in our study, the sample size for the rs-fMRI analysis could be acceptable. For the moderation analysis, we performed sample size estimation for the multiple regression analysis using G*Power (58) with the following parameters: number of predictors = 3, power = 0.80, *α* = 0.05 and small (f^2^ = 0.02), medium (f^2^ = 0.15) or large (f^2^ = 0.35) effect sizes. Estimated sample size for small, medium, and large effect sizes were 550, 77, and 36, respectively, indicating that the sample size for the moderation analysis may not be satisfactory, and the results of moderation analysis should be interpreted with caution. Third, we only had IQ data or dietary records at a single point in time. Previous studies have shown that the limited malleability of IQ by schooling and/or training (59) and IQ in early childhood are significant predictors of IQ through middle childhood into early adolescence (60). While other studies have suggested that the largest increase in human IQ is observed from 2 to 12 years, and by the age of 19–20, IQ reaches its maximum (61), and during this period, the effect of education on IQ development is observed (62). Thus, changes in IQ and nutrition intake over development should be considered in future studies to gain an in-depth understanding of associations across nutrient intake, neural development, and intelligence. Lastly, because of the age difference between the measurement of dietary habits (at age 14) and intelligence (at age 12), the current study could not examine the possibility that decreased inhibitory control, which is associated with lower general intelligence (63), could lead to increased consumption of an appetitive diet high in fat and sugar. Rodent studies consistently demonstrate that a diet high in fat and sugar can impair cognitive function, including memory (64) and spatial learning (65). Furthermore, considering the findings of a prior study that identified leaving the parental home and completing education as significant factors in altering dietary habits (29), it is unlikely that there will be a significant difference in the dietary habits of 12-year-old children (measured for intelligence) and 14-year-old children (measured for dietary habits) who typically reside at home and attend compulsory education in Japan. Thus, changing dietary habits between the ages of 12 and 14 is unlikely to have a significant impact on intelligence or cognitive function. However, to gain a complete understanding of the relationship between nutrition intake, intelligence, and neural development, further research is necessary.

In summary, our study examined the association between macronutrient intake, intelligence, and neural development in adolescents. Protein intake was positively associated with IQ in boys, whereas carbohydrate intake was negatively associated with IQ in girls in a large-cohort sample. Graph theory analysis of rs-fMRI data derived from a subsample of the large-cohort sample demonstrated that macronutrient intake was positively correlated with neural integration in the middle temporal gyrus in boys, but not in girls. Moreover, the impact of neural integration during development in the middle temporal gyrus on general intelligence differed depending on the level of macronutrient intake in boys. These results suggest that macronutrient intake in early adolescents influences neural development related to intelligence, and such influence of nutrition intake differs between boys and girls. Appropriate nutrition intake is therefore a key factor in healthy neural and cognitive development.

## Data availability statement

The original contributions presented in the study are included in the article/supplementary material, further inquiries can be directed to the corresponding author.

## Ethics statement

The studies involving humans were approved by Tokyo Metropolitan Institute of Medical Science (approval number: 12–35); The University of Tokyo (10057); and SOKENDAI (The Graduate University for Advanced Studies; 2012002). The studies were conducted in accordance with the local legislation and institutional requirements. Written informed consent for participation in this study was provided by the participants’ legal guardians/next of kin.

## Author contributions

YN: Conceptualization, Formal analysis, Investigation, Project administration, Supervision, Writing – original draft, Writing – review & editing. SY: Data curation, Funding acquisition, Writing – review & editing. NO: Data curation, Funding acquisition, Writing – review & editing. SA: Data curation, Funding acquisition, Writing – review & editing. AN: Data curation, Funding acquisition, Writing – review & editing. KK: Data curation, Funding acquisition, Writing – review & editing. SK: Data curation, Funding acquisition, Writing – review & editing.

## Footnotes

^1^For a detailed description, see https://web.conn-toolbox.org/fmri-methods/connectivity-measures/graphs-roi-level.

## References

[1] Luna B Marek S Larsen B Tervo-Clemmens B Chahal R. An integrative model of the maturation of cognitive control. Annu Rev Neurosci. (2015) 38:151–70. doi: 10.1146/annurev-neuro-071714-034054, PMID: 26154978 PMC5661874

[2] Rosenberg MD Casey BJ Holmes AJ. Prediction complements explanation in understanding the developing brain. Nat Commun. (2018) 9:589. doi: 10.1038/s41467-018-02887-9, PMID: 29467408 PMC5821815

[3] Khundrakpam BS Lewis JD Zhao L Chouinard-Decorte F Evans AC. Brain connectivity in normally developing children and adolescents. Neuro Image. (2016) 134:192–203. doi: 10.1016/j.neuroimage.2016.03.062 27054487

[4] Wu K Taki Y Sato K Hashizume H Sassa Y Takeuchi H. Topological Organization of Functional Brain Networks in healthy children: differences in relation to age, sex, and intelligence. PLoS One. (2013) 8:e55347. doi: 10.1371/journal.pone.0055347, PMID: 23390528 PMC3563524

[5] Gur RE Gur RC. Sex differences in brain and behavior in adolescence: findings from the Philadelphia neurodevelopmental cohort. Neurosci Biobehav Rev. (2016) 70:159–70. doi: 10.1016/j.neubiorev.2016.07.035, PMID: 27498084 PMC5098398

[6] Tomasi D Volkow ND. Measures of brain connectivity and cognition by sex in us children. JAMA Netw Open. (2023) 6:e230157. doi: 10.1001/jamanetworkopen.2023.0157, PMID: 36809470 PMC9945095

[7] Simmonds DJ Hallquist MN Asato M Luna B. Developmental stages and sex differences of White matter and behavioral development through adolescence: a longitudinal diffusion tensor imaging (Dti) study. NeuroImage. (2014) 92:356–68. doi: 10.1016/j.neuroimage.2013.12.044, PMID: 24384150 PMC4301413

[8] Naveed S Lakka T Haapala EA. An overview on the associations between health behaviors and brain health in children and adolescents with special reference to diet quality. Int J Environ Res Public Health. (2020) 17:953. doi: 10.3390/ijerph17030953, PMID: 32033054 PMC7037721

[9] Li J O’Connell AA. Obesity, high-calorie food intake, and academic achievement trends among U.S. school children. J Educ Res. (2012) 105:391–403. doi: 10.1080/00220671.2011.646359

[10] Ferschmann L Bos MGN Herting MM Mills KL Tamnes CK. Contextualizing adolescent structural brain development: environmental determinants and mental health outcomes. Curr Opin Psychol. (2022) 44:170–6. doi: 10.1016/j.copsyc.2021.09.014, PMID: 34688028

[11] Costello SE Geiser E Schneider N. Nutrients for executive function development and related brain connectivity in school-aged children. Nutr Rev. (2021) 79:1293–306. doi: 10.1093/nutrit/nuaa134, PMID: 33355357

[12] Stadterman J Belthoff K Han Y Kadesh AD Yoncheva Y Roy AK. A preliminary investigation of the effects of a Western diet on hippocampal volume in children. Front Pediatr. (2020) 8:58. doi: 10.3389/fped.2020.00058, PMID: 32195211 PMC7062798

[13] Cohen JF Gorski MT Gruber SA Kurdziel LB Rimm EB. The effect of healthy dietary consumption on executive cognitive functioning in children and adolescents: a systematic review. Br J Nutr. (2016) 116:989–1000. doi: 10.1017/s0007114516002877, PMID: 27487986

[14] Agarwal K Manza P Tejeda HA Courville AB Volkow ND Joseph PV. Risk assessment of maladaptive behaviors in adolescents: nutrition, screen time, prenatal exposure, childhood adversities-adolescent brain cognitive development study. J Adolesc Health. (2023) (In press). doi: 10.1016/j.jadohealth.2023.08.033 PMC10999504 37804305

[15] Mou Y Blok E Barroso M Jansen PW White T Voortman T. Dietary patterns, brain morphology and cognitive performance in children: results from a prospective population-based study. Eur J Epidemiol. (2023) 38:669–87. doi: 10.1007/s10654-023-01012-5, PMID: 37155025 PMC10232626

[16] Wang T Cao S Li D Chen F Jiang Q Zeng J. Association between dietary patterns and cognitive ability in Chinese children aged 10–15 years: evidence from the 2010 China family panel studies. BMC Public Health. (2021) 21:2212. doi: 10.1186/s12889-021-12209-2, PMID: 34863128 PMC8642971

[17] Kim JY Kang SW. Relationships between dietary intake and cognitive function in healthy Korean children and adolescents. J Lifestyle Med. (2017) 7:10–7. doi: 10.15280/jlm.2017.7.1.10, PMID: 28261556 PMC5332116

[18] Stinson EJ Piaggi P Ibrahim M Venti C Krakoff J Votruba SB. High fat and sugar consumption during ad libitum intake predicts weight gain. Obesity (Silver Spring). (2018) 26:689–95. doi: 10.1002/oby.22124, PMID: 29504262 PMC5866204

[19] Mamrot P Hanć T. The Association of the Executive Functions with overweight and obesity indicators in children and adolescents: a literature review. Neurosci Biobehav Rev. (2019) 107:59–68. doi: 10.1016/j.neubiorev.2019.08.021, PMID: 31470031

[20] Colom R Karama S Jung RE Haier RJ. Human intelligence and brain networks. Dialogues Clin Neurosci. (2010) 12:489–501. doi: 10.31887/DCNS.2010.12.4/rcolom, PMID: 21319494 PMC3181994

[21] Vértes PE. Computational models of typical and atypical brain network development. Biol Psychiatry. (2023) 93:464–70. doi: 10.1016/j.biopsych.2022.11.012, PMID: 36593135

[22] Liao X Vasilakos AV He Y. Small-world human brain networks: perspectives and challenges. Neurosci Biobehav Rev. (2017) 77:286–300. doi: 10.1016/j.neubiorev.2017.03.018, PMID: 28389343

[23] Farahani FV Karwowski W Lighthall NR. Application of graph theory for identifying connectivity patterns in human brain networks: a systematic review. Front Neurosci. (2019) 13:585. doi: 10.3389/fnins.2019.00585, PMID: 31249501 PMC6582769

[24] Ando S Nishida A Yamasaki S Koike S Morimoto Y Hoshino A. Cohort profile: the Tokyo Teen cohort study (TTC). Int J Epidemiol. (2019) 48:1414-g. doi: 10.1093/ije/dyz033, PMID: 30879075 PMC6857749

[25] Okada N Ando S Sanada M Hirata-Mogi S Iijima Y Sugiyama H. Population-neuroscience study of the Tokyo Teen cohort (Pn-Ttc): cohort longitudinal study to explore the neurobiological substrates of adolescent Psychological and behavioral development. Psychiatry Clin Neurosci. (2019) 73:231–42. doi: 10.1111/pcn.12814, PMID: 30588712

[26] Wechsler D Psychological C. WISC-iii: Wechsler intelligence scale for children: Manual. 3rd ed. San Antonio: Psychological Corp. San Antonio (1991).

[27] Kobayashi S Murakami K Sasaki S Okubo H Hirota N Notsu A. Comparison of relative validity of food group intakes estimated by comprehensive and brief-type self-administered diet history questionnaires against 16 D dietary Records in Japanese Adults. Public Health Nutr. (2011) 14:1200–11. doi: 10.1017/s1368980011000504, PMID: 21477414

[28] Okuda M Sasaki S Bando N Hashimoto M Kunitsugu I Sugiyama S. Carotenoid, tocopherol, and fatty acid biomarkers and dietary intake estimated by using a brief self-administered diet history questionnaire for older Japanese children and adolescents. J Nutr Sci Vitaminol. (2009) 55:231–41. doi: 10.3177/jnsv.55.231, PMID: 19602831

[29] Winpenny EM van Sluijs EMF White M Klepp K-I Wold B Lien N. Changes in diet through adolescence and early adulthood: longitudinal trajectories and association with key life transitions. Int J Behav Nutr Phys Act. (2018) 15:86. doi: 10.1186/s12966-018-0719-8 30200990 PMC6131755

[30] Dennis E Manza P Volkow ND. Socioeconomic status, Bmi, and brain development in children. Transl Psychiatry. (2022) 12:33. doi: 10.1038/s41398-022-01779-3, PMID: 35075111 PMC8786961

[31] de Onis M Onyango AW Borghi E Siyam A Nishida C Siekmann J. Development of a who growth reference for school-aged children and adolescents. Bull World Health Organ. (2007) 85:660–7. doi: 10.2471/blt.07.043497, PMID: 18026621 PMC2636412

[32] Tomasi D Volkow ND. Associations of family income with cognition and brain structure in USA children: prevention implications. Mol Psychiatry. (2021) 26:6619–29. doi: 10.1038/s41380-021-01130-0, PMID: 33990770 PMC8590701

[33] Nieto-Castanon A Whitfield-Gabrieli S. Conn functional connectivity toolbox: Rrid Scr_009550, Release 22. Boston, MA: Hilbert Press (2022).

[34] Behzadi Y Restom K Liau J Liu TT. A component based noise correction method (Compcor) for Bold and perfusion based Fmri. NeuroImage. (2007) 37:90–101. doi: 10.1016/j.neuroimage.2007.04.042, PMID: 17560126 PMC2214855

[35] Schaefer A Kong R Gordon EM Laumann TO Zuo XN Holmes AJ. Local-global Parcellation of the human cerebral cortex from intrinsic functional connectivity MRI. Cereb Cortex. (2018) 28:3095–114. doi: 10.1093/cercor/bhx179, PMID: 28981612 PMC6095216

[36] Luo L Xiao M Luo Y Yi H Dong D Liu Y. Knowing what you feel: inferior frontal gyrus-based structural and functional neural patterns underpinning adaptive body awareness. J Affect Disord. (2022) 315:224–33. doi: 10.1016/j.jad.2022.07.051, PMID: 35901991

[37] Jessen F Flacke S Granath DO Manka C Scheef L Papassotiropoulos A. Encoding and retrieval related cerebral activation in continuous verbal recognition. Brain Res Cogn Brain Res. (2001) 12:199–206. doi: 10.1016/s0926-6410(01)00046-5, PMID: 11587890

[38] Kong R Yang Q Gordon E Xue A Yan X Orban C. Individual-specific areal-level Parcellations improve functional connectivity prediction of behavior. Cereb Cortex. (2021) 31:4477–500. doi: 10.1093/cercor/bhab101, PMID: 33942058 PMC8757323

[39] Shi J Huang H Jiang R Mao X Huang Q Li A. The right inferior frontal gyrus plays an important role in unconscious information processing: activation likelihood estimation analysis based on functional magnetic resonance imaging. Front Neurosci. (2022) 16:781099. doi: 10.3389/fnins.2022.781099, PMID: 35401077 PMC8987111

[40] Wang Y Chiew V. On the cognitive process of human problem solving. Cogn Syst Res. (2010) 11:81–92. doi: 10.1016/j.cogsys.2008.08.003

[41] Leech R Sharp DJ. The role of the posterior cingulate cortex in cognition and disease. Brain. (2013) 137:12–32. doi: 10.1093/brain/awt162, PMID: 23869106 PMC3891440

[42] Leech R Smallwood J. The posterior cingulate cortex: insights from structure and function. Handb Clin Neurol. (2019) 166:73–85. doi: 10.1016/b978-0-444-64196-0.00005-4 31731926

[43] Rolls ET Wirth S Deco G Huang CC Feng J. The human posterior cingulate, Retrosplenial, and medial parietal cortex effective connectome, and implications for memory and navigation. Hum Brain Mapp. (2023) 44:629–55. doi: 10.1002/hbm.26089, PMID: 36178249 PMC9842927

[44] Rolls ET. The cingulate cortex and limbic Systems for Emotion, action, and memory. Brain Struct Funct. (2019) 224:3001–18. doi: 10.1007/s00429-019-01945-2, PMID: 31451898 PMC6875144

[45] Utevsky AV Smith DV Huettel SA. Precuneus is a functional Core of the default-mode network. J Neurosci. (2014) 34:932–40. doi: 10.1523/jneurosci.4227-13.2014, PMID: 24431451 PMC3891968

[46] Yeo BT Krienen FM Sepulcre J Sabuncu MR Lashkari D Hollinshead M. The Organization of the Human Cerebral Cortex Estimated by intrinsic functional connectivity. J Neurophysiol. (2011) 106:1125–65. doi: 10.1152/jn.00338.2011, PMID: 21653723 PMC3174820

[47] Cavanna AE Trimble MR. The Precuneus: a review of its functional anatomy and behavioural correlates. Brain. (2006) 129:564–83. doi: 10.1093/brain/awl004 16399806

[48] Huijbers W Schultz AP Vannini P McLaren DG Wigman SE Ward AM. The encoding/retrieval Flip: interactions between memory performance and memory stage and relationship to intrinsic cortical networks. J Cogn Neurosci. (2013) 25:1163–79. doi: 10.1162/jocn_a_00366, PMID: 23384193 PMC3730829

[49] Heckner MK Cieslik EC Eickhoff SB Camilleri JA Hoffstaedter F Langner R. The aging brain and executive functions revisited: implications from Meta-analytic and functional-connectivity evidence. J Cogn Neurosci. (2021) 33:1716–52. doi: 10.1162/jocn_a_01616, PMID: 32762523 PMC8630425

[50] Santarnecchi E Galli G Polizzotto NR Rossi A Rossi S. Efficiency of weak brain connections support general cognitive functioning. Hum Brain Mapp. (2014) 35:4566–82. doi: 10.1002/hbm.22495, PMID: 24585433 PMC6869093

[51] Dixon ML De La Vega A Mills C Andrews-Hanna J Spreng RN Cole MW. Heterogeneity within the Frontoparietal control network and its relationship to the default and dorsal attention networks. Proc Natl Acad Sci. (2018) 115:E1598–607. doi: 10.1073/pnas.1715766115, PMID: 29382744 PMC5816169

[52] Xu J Wang J Fan L Li H Zhang W Hu Q. Tractography-based Parcellation of the human middle temporal gyrus. Sci Rep. (2015) 5:18883. doi: 10.1038/srep18883, PMID: 26689815 PMC4686935

[53] Briggs RG Tanglay O Dadario NB Young IM Fonseka RD Hormovas J. The unique Fiber anatomy of middle temporal gyrus default mode connectivity. Operative Neurosurg. (2021) 21:E8–E14. doi: 10.1093/ons/opab109, PMID: 33929019 PMC8203421

[54] Beaty RE Cortes RA Zeitlen DC Weinberger AB Green AE. Functional realignment of Frontoparietal subnetworks during divergent creative thinking. Cereb Cortex. (2021) 31:4464–76. doi: 10.1093/cercor/bhab100, PMID: 33895837

[55] Schmithorst VJ. Developmental sex differences in the relation of neuroanatomical connectivity to intelligence. Intelligence. (2009) 37:164–73. doi: 10.1016/j.intell.2008.07.001 21297966 PMC3032174

[56] Weiss EM Kemmler G Deisenhammer EA Fleischhacker WW Delazer M. Sex differences in cognitive functions. Personal Individ Differ. (2003) 35:863–75. doi: 10.1016/S0191-8869(02)00288-X

[57] Benito-León J Sanz-Morales E Melero H Louis ED Romero JP Rocon E. Graph theory analysis of resting-state functional magnetic resonance imaging in essential tremor. Hum Brain Mapp. (2019) 40:4686–702. doi: 10.1002/hbm.24730, PMID: 31332912 PMC6865733

[58] Faul F Erdfelder E Lang AG Buchner A. G*power 3: a flexible statistical power analysis program for the social, behavioral, and biomedical sciences. Behav Res Methods. (2007) 39:175–91. doi: 10.3758/bf03193146, PMID: 17695343

[59] Herrnstein RJ Murray C. The bell curve: Intelligence and class structure in American life. New York, NY: Simon and Schuster (2010).

[60] Zhu Z Chang S Cheng Y Qi Q Li S Elhoumed M. Early life cognitive development trajectories and intelligence quotient in middle childhood and early adolescence in rural Western China. Sci Rep. (2019) 9:18315. doi: 10.1038/s41598-019-54755-1, PMID: 31797987 PMC6892923

[61] Volkova EV. Age dynamics of intelligence in adolescence and early adulthood. Procedia Soc Behav Sci. (2014) 140:440–6. doi: 10.1016/j.sbspro.2014.04.450

[62] Brinch CN Galloway TA. Schooling in adolescence raises IQ scores. Proc Natl Acad Sci. (2012) 109:425–30. doi: 10.1073/pnas.1106077109 22203952 PMC3258640

[63] Kang W Hernández SP Rahman MS Voigt K Malvaso A. Inhibitory control development: a network neuroscience perspective. Front Psychol. (2022) 13:651547. doi: 10.3389/fpsyg.2022.651547, PMID: 36300046 PMC9588931

[64] Reichelt AC Gibson GD Abbott KN Hare DJ. A high-fat high-sugar diet in adolescent rats impairs social memory and alters chemical markers characteristic of atypical neuroplasticity and Parvalbumin interneuron depletion in the medial prefrontal cortex. Food Funct. (2019) 10:1985–98. doi: 10.1039/c8fo02118j, PMID: 30900711

[65] Abbott KN Arnott CK Westbrook RF Tran DMD. The effect of high fat, high sugar, and combined high fat-high sugar diets on spatial learning and memory in rodents: a meta-analysis. Neurosci Biobehav Rev. (2019) 107:399–421. doi: 10.1016/j.neubiorev.2019.08.010, PMID: 31454627

